# Alteration in forward model prediction of sensory outcome of motor action in focal hand dystonia

**DOI:** 10.3389/fnhum.2013.00172

**Published:** 2013-07-19

**Authors:** André Lee, Shinichi Furuya, Matthias Karst, Eckart Altenmüller

**Affiliations:** ^1^Institute for Music Physiology and Musicians' Medicine, University for MusicDrama and Media Hannover, Hannover Germany; ^2^Department of Anesthesiology, Pain Clinic, Hannover Medical SchoolHannover, Germany

**Keywords:** dystonia, internal models, sensory prediction, musicians, cerebellum

## Abstract

Focal hand dystonia in musicians is a movement disorder affecting highly trained movements. Rather than being a pure motor disorder related to movement execution only, movement planning, error prediction, and sensorimotor integration are also impaired. Internal models (IMs), of which two types, forward and inverse models have been described and most likely processed in the cerebellum, are known to be involved in these tasks. Recent results indicate that the cerebellum may be involved in the pathophysiology of focal dystonia (FD). Thus, the aim of our study was to investigate whether an IM deficit plays a role in FD. We focused on the forward model (FM), which predicts sensory consequences of motor commands and allows the discrimination between external sensory input and input deriving from motor action. We investigated 19 patients, aged 19–59 and 19 healthy musicians aged 19–36 as controls. Tactile stimuli were applied to fingers II–V of both hands by the experimenter or the patient. After each stimulus the participant rated the stimulus intensity on a scale between 0 (no sensation) and 1 (maximal intensity). The difference of perceived intensity between self- and externally applied (EA) stimuli was then calculated for each finger. For assessing differences between patients and controls we performed a cluster analysis of the affected hand and the corresponding hand of the controls using the fingers II–V as variables in a 4-dimensional hyperspace (chance level = 0.5). Using a cluster analysis, we found a correct classification of the affected finger in 78.9–94.7%. There was no difference between patients and healthy controls of the absolute value of the perceived stimulus intensity. Our results suggest an altered FM function in focal hand dystonia. It has the potential of suggesting a neural correlate within the cerebellum and of helping integrate findings with regard to altered sensorimotor processing and altered prediction in FD in a single framework.

## Introduction

Musicians' dystonia (MD), a form of focal dystonia (FD), is characterized by a loss of fine motor control of highly trained and automated movements that severely impairs the playing ability and often threatens the professional career.

While the phenomenology of MD might imply that it is a pure disorder of movement execution, it has been shown that sensorimotor integration (Wu et al., 2009), movement preparation (Hallett, 2000; Lim et al., 2004), and error prediction (Ruiz et al., 2009) are altered. A recent study demonstrated that the prediction of temporal characteristics is also impaired in writer's cramp (Avanzino et al., 2013). These findings indicate that mechanisms involved in feed forward motor prediction are compromised.

Music making relies strongly on predictive mechanisms, as was shown by Ruiz and colleagues in an EEG-study in healthy pianists. When playing trained musical excerpts on a piano, they found a negative component 70 ms prior to an erroneous action—termed pre-error negativity—which originated in the anterior cingulate cortex (Ruiz et al., 2009), an area that has been described before as being involved in internal model (IM) prediction (Blakemore et al., 1998; Boecker et al., 2005). The same error prediction mechanism was shown to be abnormal in MD (Ruiz et al., 2011) pointing at altered predictive mechanisms.

One neural mechanism that is involved in feed forward control and motor preparation of highly automated movements as well as sensorimotor integration are IMs (Wolpert and Kawato, 1998; Haruno et al., 2001), of which two types have been described: one predicting sensory consequences of motor commands (forward models, FMs) and another one generating motor commands for a desired consequence (inverse models). Neuronal networks processing IMs are most likely located in the cerebellum (Wolpert et al., 1998; Imamizu et al., 2000; Kawato et al., 2003; Boecker et al., 2005; Ito, 2008; Imamizu and Kawato, 2012), which is known to be involved in movement preparation (Purzner et al., 2007). From functional (Odergren et al., 1998; Preibisch et al., 2001) or structural imaging (Pizoli et al., 2002) as well as from lesion studies (Krauss et al., 1997; Le Ber et al., 2006; Zadro et al., 2008) and animal models (Pizoli et al., 2002) there is increasing evidence for an involvement of the cerebellum in the pathophysiology of dystonias as well (Avanzino and Abbruzzese, 2012; Sadnicka et al., 2012) and it has been argued that dystonia may result from a dysfunctional network including the cerebellum and the basal ganglia (Jinnah and Hess, 2006; Neychev et al., 2008; Argyelan et al., 2009).

The aim of the study was to investigate, whether an altered function of FM plays a role in MD. We thus chose a paradigm that specifically addresses FM-function. It has been shown that healthy subjects perceive externally applied stimuli (EA) more intensely than identical self applied (SA) tactile stimuli. This phenomenon has been repeatedly investigated (Weiskrantz et al., 1971; Blakemore et al., 1998, 1999, 2000, 2001; Karst et al., 2005; Pridmore et al., 2006) and explained by FM, which receive an efference copy of a motor command and predict the sensory consequence (Wolpert and Kawato, 1998; Wolpert et al., 1998; Kawato et al., 2003). If the predicted sensory consequence is similar or identical to the actual sensory feedback, the sensation is attenuated. Thereby we are able to distinguish between potentially significant external sensory input and sensory input arising as a mere consequence of our own actions. Thus, by applying EA and SA, FM-function may be addressed. Our paradigm does not allow for a direct investigation of the cerebellum. However, the role of the cerebellum for FM has been confirmed in two imaging studies by Blakemore et al. (1998, 2001) so that an altered FM-function may allude an involvement of the cerebellum being part of a larger network including the basal ganglia and parts of the cortex. Since MD may affect single fingers, we assessed individual fingers separately, differentiating between dystonic and non-dystonic fingers.

Our hypothesis was that an altered FM-function at the affected fingers enables a detection of the affected fingers when comparing the differential value of EA and SA. Furthermore, we were interested in whether, if a FM-deficit exists, the amount of deficit can be correlated to epidemiological parameters as MD-onset, MD-duration, MD-severity, or the course of the disease.

## Methods

Nineteen patients (age 19–59, mean 40.8, SD 13.0) with MD of the fingers (patients) (Table 1), and 19 healthy musicians (age 19–36, mean 24.6, SD 4.1) matched for instrument, handedness, and gender (controls) participated in the experiment. We acknowledge the age-difference, however, it was shown before with the identical stimulus device we used that neither perception intensity nor difference between EA or SA is related to this variable (Pridmore et al., 2006). Still age cannot be ruled out as a potential influencing factor. The same holds true for the two left-handers included, who were, however, matched in the control group. Since all participants were professional musicians or students, all had similar amounts of playing activity in the months prior to the investigation. Seven patients had received Botulinum Toxin with the last injection being at least 3 months prior to the investigation, thereby minimizing a potential influence of this treatment. With regard to tactile performance it is known that piano-playing leads to a superior two-point discrimination when compared to non-musicians (Ragert et al., 2004). However, we included only professional musicians in both groups. Furthermore, our paradigm differed from a two-point discrimination task, which will be discussed in detail below.

**Table 1:** **Epidemiological data of the patients**.

**Participant**	**M/W**	**Affected side**	**Age at onset**	**Duration of dystonia (years)**	**Dystonia severity at symptom onset**	**Playing ability today**	**Difference in playing ability**	**Handedness**
1	M	l	39	6	50%	60%	10%	l
2	M	l	31	9	90%	85%	−5%	r
3	M	r	52	0	50%	70%	20%	r
4	M	l	17	2	70%	60%	−10%	r
5	M	l/r	26	29	40%	60%	20%	r
6	W	l	28	16	80%	65%	−15%	r
7	W	l	33	7	70%	85%	15%	r
8	W	l	25	4	90%	60%	−30%	r
9	M	r	31	3	60%	70%	10%	r
10	M	l	37	7	90%	60%	−30%	l
11	M	r	52	4	70%	80%	10%	r
12	M	r	56	2	50%	60%	10%	r
13	W	r	21	2	20%	30%	10%	r
14	M	r	41	11	80%	20%	−60%	r
15	W	l	21	1	35%	53%	18%	r
16	W	r	21	2	85%	70%	−15%	r
17	M	r	29	7	99%	94%	−5%	r
18	M	l	47	12	65%	65%	0%	r
19	M	r	32	8	80%	65%	−15%	r
Mean			33.6	6.9	67.1	63.8	−3.3	
SD			11.6	6.8	21.5	17.41	20.9	

The study was approved by the local ethics committee and written informed consent was obtained from each participant. The study was conducted in accordance with the declaration of Helsinki.

**Figure 1:**
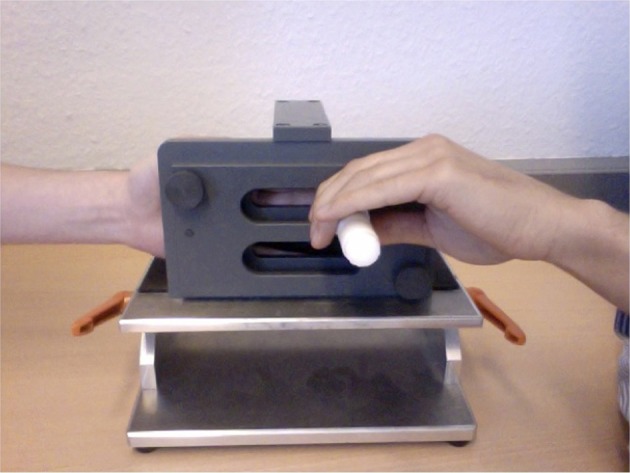
**The stimulating device**. The left hand is inside the stimulator. The experimenter's left hand (for EA) (or the participant's right hand for SA) is moving a white plastic pointer with a spherical tip of 1 mm diameter which is counterweighted with a spring to maintain a constant pressure of 0.17 N at the stimulated finger. The device is designed in a way that the tip touches the palmar side of only one finger perpendicularly.

Stimuli were applied via a stimulus device (Figure 1) (Karst et al., 2005) which had a plastic pointer with a spherical tip of 1 mm diameter. The tip was counterweighted with a spring to maintain a constant pressure of 0.17 N at the stimulated finger. For stimulation the participant placed his/her hand on the hand-rest of the device in a way that the pointer could only move in a straight line perpendicular to the palmar surface of the stimulated finger. At rest, the handle for moving the pointer did not produce any noise and for stimulus onset could be touched in a way that no noise was generated until movement actually started. Thus, an auditory clue for the participant indicating stimulus onset could be excluded. Fingers II–V were stimulated separately in a randomized order to account for a sequence effect. Each finger was stimulated eight times, either in the order SA–EA or vice versa, again in a randomized order. Thus, 16 stimulations were obtained per finger for each participant. One stimulus consisted in one back and forth movement of the pointer. To assure a constant movement speed the movement was paced by a metronome set to 60 bpm with 2 s per stimulus. The perceived tactile sensation was assessed after each stimulus on a visual analog rating scale between 0 (no sensation) and 1 (extremely intense sensation). Participants were asked to maintain their scale throughout the experiment. Before each stimulus, participants were asked to close their eyes in order to exclude a prediction of stimulus onset from visual clues for EA. The differential value between the perceived tactile sensation of SA and EA was then calculated for each pair of stimuli (EA–SA) for each finger of each hand. The value was then logarithmic-transformed and standardized within a participant.

In order to determine effects of FD on the perceptual predictability of the hand, a cluster analysis using support vector machine (SVM) was performed to datasets of each hand for all controls and patients in a four dimensional hyperspace (i.e., four fingers). A binary classification by SVM identifies whether the datasets that represent differential values between SA and EA conditions at the four fingers can be segregated in the hyperspace according to if one of the fingers is affected (Vapnik, 1995). A rationale of using the differential value as an input of the cluster analysis is that this value should be close to 0 if an individual fails to predict sensory outcome elicited by the self-motion (i.e., FM). This analysis was performed for each of the fingers and for each of the hands separately. To evaluate the predictability of a classifier, a leave-one-out cross-validation (LOOCV) was performed using a custom-made script written by MATLAB (Mathworks co.). In a LOOCV, each dataset is treated as the testing dataset only once, and serves as the training dataset *N*–1 times, where *N* is the total number of datasets (subjects), and therefore the parameters need to be tuned *N* times. This yields the number of misclassified testing dataset, which was divided by the total number of datasets and then subtracted from 1. This value was defined as the LOOCV score that represents whether the datasets can be classified by presence or absence of FD (chance level = 0.5).

We further assessed age at MD onset, MD-duration and severity (after MD-onset and today); playing ability (after MD-onset and today) and the course of the disease with a questionnaire previously used for this purpose (Jabusch et al., 2005).

Patients assessed their playing ability on a scale from 0 (unable to play) to 100% (no impairment). The course of the disease was then calculated as the difference between the playing ability at now and at MD-onset, with a negative value meaning deterioration, a positive value improvement and no difference and unchanged symptoms, respectively. MD-severity was defined by the investigator according to the subjective playing ability with a score of 80–100% signifying mild MD, 60–79% moderate MD, and <60% severe MD.

To assess whether FD influences mere perception evoked by the external stimulus and predictability of perceptive outcome evoked by the self-motion, Three-Way analysis of variance (ANOVA) of a mixed design was performed using finger (index, middle, ring, and little fingers, 4 factors) and hand (right and left, 2 factors) as within-group independent variables and using group (healthy and dystonia) as a between-group independent variable.

## Results

MD-onset occurred at an age of (mean ± SD) 33.6 ± 11.6 years with a MD-duration of 6.95 ± 6.8 years. Playing ability was 67.1 ± 21.5% at MD-onset and 63.8 ± 17.4% at present. Difference between present playing ability and playing ability at onset was −3.3 ± 20.9% (Table 1).

**Figure 2:**
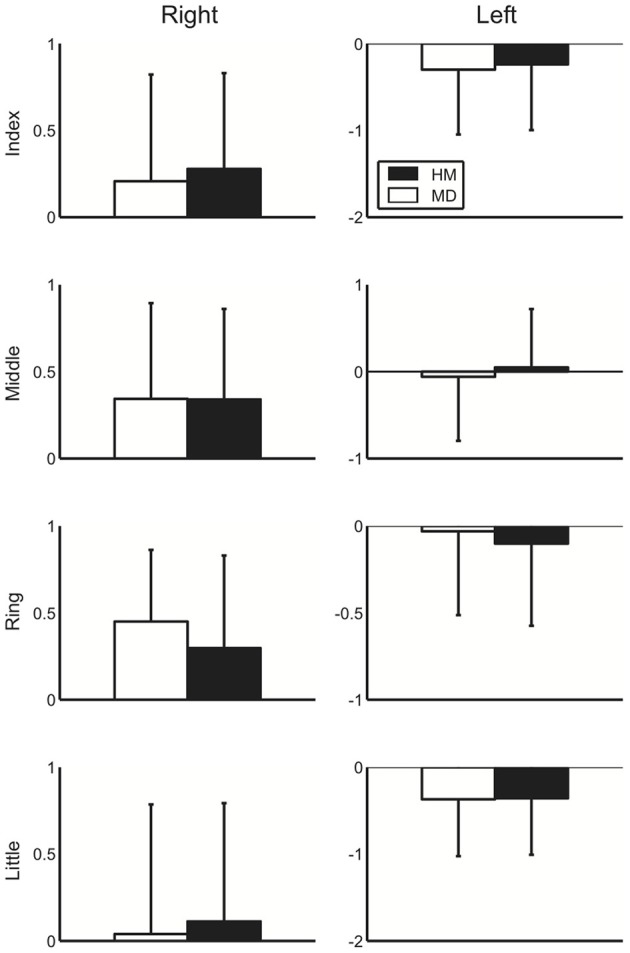
**A group mean of the logarithmic-transformed and standardized perceptual value at the external stimulus condition at each of the four fingers at the right (left panel) and left (right panel) hands across healthy musicians (HM) and musicians dystonia (MD)**. Error bar indicates one standard deviation across participants.

Figure 2 displays the group mean of the logarithmic-transformed and standardized value of perception elicited by external stimuli across healthy musicians and musicians with dystonia for all fingers in both hands. Here, no apparent group difference could be shown. To assess a potential difference in perception elicited by external stimuli between the healthy musicians and dystonia patients, a Three-Way mixed design ANOVA was performed. The result showed no main effect of group [*F*_(1, 36)_ = 0.001, *p* = 0.97], which confirmed no difference in perception evoked by an external stimulus. Similarly, Three-Way mixed design ANOVA was performed to differential values between the SA and EA conditions. Again, there was no significant main effect of group [*F*_(1, 36)_ = 0.92, *p* = 0.34], which indicates no effect of FD on predictability of perceptional outcome elicited by the self-motion at each of the four fingers for both hands. In order to assess the effect of past injection of Botulinum Toxin on perception evoked by an external stimulus, the patients were divided into two groups according to whether there was a previous treatment with Botulinum Toxin or not. Then a Three-Way ANOVA was performed only with the patient group. There was no significant main effect of the injection [*F*_(1, 17)_ = 1.2, *p* = 0.29], which confirmed no effect of past injection of Botulinum Toxin on the absolute perception.

In order to further explore the possibility that focal hand dystonia influences the predictability of tactile stimulation not of a single affected finger, but of a combination of multiple fingers in a specific manner, datasets of all four fingers were inputted into a cluster analysis that identifies whether a finger is affected. The LOOCV revealed a correct classification rate ranging from 78.9 to 94.7%. For the right hand, correct classification rate of whether a certain finger is affected based on datasets of the four fingers was 94.7, 94.7, and 86.8% at the index, middle, and ring finger, respectively. Note that we did not have a patient with an affected little finger of the right hand. For the left hand, correct classification rate was 94.7, 89.5, 78.9, and 84.2% at the index, middle, ring, and little finger, respectively (Table 2). The findings of ANOVA and cluster analysis indicated that focal hand dystonia altered the perceptual predictability of not a single finger but multiple fingers of the affected hand in a complex manner, which will be discussed in the discussion part in detail.

**Table 2:** **Results of the clustering analysis in the 4-dimensional space with four fingers as variables**.

	**Index**	**Middle**	**Ring**	**Little**
Right	0.947	0.947	0.868	NaN
Left	0.947	0.895	0.789	0.842

Identification of the affected finger was successful in 78.9–94.7% (chance level = 50%). Note that none of the patients had a dystonia at the 5th finger of the left hand.

A linear correlation analysis yielded no significant correlation between the differential value between the two conditions and any of the epidemiological parameters (*p* > 0.05).

## Discussion

It is known that single-finger individuation and independence is more pronounced in musicians as compared to non-musicians (Furuya et al., 2011). At the same time, with regard to motor symptoms in MD as well as in WC, more than one single finger may be affected. With regard to the somatosensory system, a recent study could show that in FD alterations in the somatosensory cortex (area 3b) occur digit-specific for those digits affected by dystonia (Nelson et al., 2009), underlining the importance of single finger investigation when addressing the somatosensory system in FD. For this reason, our hypothesis was that alterations in FM function may be detected at a single finger level. Hence, we chose a paradigm that allowed us to assess differences between affected and unaffected fingers separately. This resulted in a four-dimensional hyperspace and we acknowledge the non-intuitive and abstract character of this analysis. However, it is apparent that FD itself is a disorder for which no straightforward pathophysiological answer has been found so far. Therefore, we assume that the complexity of the analysis is adequate to the underlying challenge. We consider findings of a recent study investigating Aristotle's illusion in FD (Tinazzi et al., 2013) as supportive for the complex relation between all fingers in FD. Aristotle's illusion describes the phenomenon that when crossing two fingers and touching a small spherical object, it feels like one is touching two objects. In the study, alteration of the illusion was more present in the fingers not affected by dystonia, yet at the same time the alteration was correlated with symptom severity (Tinazzi et al., 2013). The fact of a symptom depending on symptom severity at the affected fingers but surprisingly manifesting itself at the unaffected fingers alludes at an interaction between all fingers of a higher dimensional order. It again underlines the importance of investigating at the single finger level but considering higher dimensional analysis, i.e., the combined analysis of all four fingers of the hand. The most important finding of our work was that it was possible to correctly identify all affected fingers with an accuracy of 78.9–94.7% when using the differential values between SA and EA for the cluster analysis. This indicates an altered prediction of the sensory consequences of motor action at the affected fingers as compared to the unaffected fingers or those of healthy controls. We found a slightly lower classification for the left hand than for the right hand. Interpretation of this finding is not straightforward. However, our hypothesis was not aimed at assessing differences depending on which side is affected and thus our paradigm was not designed to answer this question. To realize it, an equal distribution of affected fingers for each hand should be investigated in a future study. Thus, an explanation of this finding remains speculative. It may be related to whether or not the dominant hand is affected. It is known that MD occurs more often at the dominant side (Baur et al., 2011) and that prognosis is significantly better if the dominant side is affected (unpublished data), which again may be discussed in the context of meta-plasticity, which has been described to be enhanced in musicians (Ragert et al., 2004). Our study was not designed to test for executive control via the cerebellum. However, it is known that the cerebellum plays a crucial role in cognitive and executive control (Koziol et al., 2010) and that cerebellar volume correlates with psychometric intelligence (Hogan et al., 2011). Thus, an impairment of executive control cannot be ruled out and should be addressed in future studies. Our finding of abnormal prediction may explain findings of impaired movement imagination in FD while movement observation is normal (Castrop et al., 2012). It is known that when comparing executed, imagined, or observed movements, neural activation in observation resembles execution least (Szameitat et al., 2012), whereas motor imagination activates neural mechanisms that are very similar to motor execution (Jeannerod, 1994; Jeannerod and Frak, 1999; Macuga and Frey, 2012). Increased activity in motor networks could be found in motor imagination compared to motor observation but not vice versa (Castrop et al., 2012; Macuga and Frey, 2012). Notably, increased activation was found (albeit not exclusively) in the cerebellum and in the anterior cingulate (Deiber et al., 1998; Macuga and Frey, 2012), both structures being involved in IM networks (Boecker et al., 2005; Ruiz et al., 2009). Following the hierarchical structure according to which imagination results in the activation of a different subset of the neuronal network of motor execution than observation (Macuga and Frey, 2012; Szameitat et al., 2012) and integrating the above findings, one may conclude that movement observation tasks rely on predictive mechanisms to a lesser extent than movement imagination tasks.

In a purely observational task the inverse model and not the FM is involved (Gazzola and Keysers, 2009). Thus, with an impaired FM-mechanism, observation should be normal in FD whereas imagination-tasks, where IMs are involved, should be impaired. Exactly this was recently found by Castrop et al. (2012). However, Gazzola and Keysers further state that once the inverse model has been triggered by movement observation, further predictions with regard to the consequences of the movement can potentially be made using the FM (Gazzola and Keysers, 2009). Thus, it should be expected that, if FM is altered, as suggested by our findings, even though observation itself is unaltered, predictions based on motor observation should be impaired in FD (since then FM would be necessary). This is exactly what Avanzino et al. (2013) found, when demonstrating that prediction of temporal consequences of an observed writing was impaired in patients with FD.

Our findings of altered prediction of consequences of motor action in MD corroborates the suggestion that representation of movements is impaired at a central level (Avanzino et al., 2013). We would like to extend this suggestion by hypothesizing that also the representation of the consequences of movements is impaired. As in our study, Avanzino et al. found no correlation between the prediction error and clinical variables such as disease duration or severity either.

There is evidence that abnormal sensory processing itself may be dissociated from abnormal motor processing. A recent study demonstrated that somatosensory alterations can be detected in the absence of motor abnormalities at rest (Weise et al., 2012). In the study mentioned above investigating Aristotle's illusion (Tinazzi et al., 2013), the findings of abnormalities in the unaffected fingers was interpreted as a dissociation between abnormal processing of sensory signals and motor impairment. Here, as mentioned above, a correlation could be shown between symptom severity and a reduction in the illusion.

One difference between this study and our study or the study by Avanzino et al. is that the former (Tinazzi et al., 2013) addressed Aristotle's illusion, which does not rely on predictive mechanisms whereas the latter studies assessed prediction mechanisms. In order to integrate the above-mentioned findings we thus hypothesize firstly that altered somatosensory integration comprising predictive mechanisms occurs independently from motor impairment and secondly that altered somatosensory integration that does not comprise predictive mechanisms is correlated to symptom severity.

We did not find a difference between patients and healthy controls with regard to the absolute value of the perceived stimulus intensity. It has been shown, however, that in FD somatosensory processing, as spatial (Bara-Jimenez et al., 2000; Sanger et al., 2001; Molloy et al., 2003) or temporal discrimination (Fiorio et al., 2008; Scontrini et al., 2009) and vibrotactile-induced illusion of movement (VIIM), (Rome and Grünewald, 1999; Frima et al., 2008) is impaired. One reason for our finding may be that our stimulus differed from the other tasks with regard to its quality and the receptors being stimulated. The spatial discrimination task consists of a one-dimensional spatial stimulus, where mainly slowly adapting (SA)-I mechanoreceptors are stimulated (Schmidt et al., 2010). In the VIIM mainly I and II afferents of muscle spindles are stimulated (Schmidt et al., 2010) and higher order processing of the stimulus (i.e., the illusion of a movement in an immobilized limb), (Goodwin et al., 1972) and not the stimulus itself, neither in its spatial nor temporal component are in the focus of interest. Finally for temporal discrimination higher order integration of the temporal aspect is considered, regardless of the spatial component. Since mostly electrical pulses are applied (Scontrini et al., 2009), it cannot be excluded, that different receptors (nociceptors as well as mechanoreceptors) were stimulated, however, it is unlikely that rapidly adapting (RA)-receptors play a major role.

In contrast to these stimuli, in our task, focus of perception was directed toward the intensity caused by the movement of the tip on the surface of the skin (rather than its pressure), a two-dimensional, spatio-temporal stimulus that is conveyed by RA-receptors (Schmidt et al., 2010). The primary aim of our paradigm was not to find differences in perception intensity but rather to compare differential values between SA and EA and investigate differences in the stimulus anticipation. Thus, our paradigm does not allow for any conclusions to be drawn with regard to how FD affects processing of afferent input from different somatosensory receptors. It is tempting, however, to hypothesize that processing of afferents from different receptors might be affected to a different extent. Further studies are needed that address this hypothesis.

## Conclusion

Our results imply that predictive function of FM is altered in MD. Thus, we propose a specific neural correlate for the pathogenesis of MD possibly located within the cerebellum that is involved in sensorimotor integration, i.e., IMs. This finding fits with the network hypothesis of dystonia (Jinnah and Hess, 2006; Neychev et al., 2008; Argyelan et al., 2009) that likely includes structures involved in FM-processing as the cerebellum, the anterior cingulate (Blakemore et al., 1998; Boecker et al., 2005), and the basal ganglia. Furthermore, FM alterations have the powerful potential to integrate findings with regard to altered sensorimotor integration and altered prediction mechanisms found in FD in one single framework.

### Conflict of interest statement

The authors declare that the research was conducted in the absence of any commercial or financial relationships that could be construed as a potential conflict of interest.
